# Non‐invasive assessment of cultivar and sex of *Cannabis sativa* L. by means of hyperspectral measurement

**DOI:** 10.1002/pei3.10116

**Published:** 2023-08-17

**Authors:** Andrea Matros, Patrick Menz, Alison R. Gill, Armando Santoscoy, Tim Dawson, Udo Seiffert, Rachel A. Burton

**Affiliations:** ^1^ ARC Centre of Excellence in Plant Energy Biology, School of Agriculture, Food and Wine University of Adelaide Adelaide South Australia Australia; ^2^ Biosystems Engineering Fraunhofer IFF Magdeburg Germany; ^3^ Advanced Seeds Australia Adelaide South Australia Australia; ^4^ Australian Hemp Seed Company Gawler South Australia Australia; ^5^ Australian Plant Phenomics Facility, School of Agriculture, Food and Wine & Waite Research Institute University of Adelaide Urrbrae South Australia Australia; ^6^ Present address: Compolytics GmbH Barleben Saxony‐Anhalt Germany

**Keywords:** cannabis, cultivar, industrial hemp, machine learning, prediction, sex, spectral measurement

## Abstract

*Cannabis sativa* L. is a versatile crop attracting increasing attention for food, fiber, and medical uses. As a dioecious species, males and females are visually indistinguishable during early growth. For seed or cannabinoid production, a higher number of female plants is economically advantageous. Currently, sex determination is labor‐intensive and costly. Instead, we used rapid and non‐destructive hyperspectral measurement, an emerging means of assessing plant physiological status, to reliably differentiate males and females. One industrial hemp (low tetrahydrocannabinol [THC]) cultivar was pre‐grown in trays before transfer to the field in control soil. Reflectance spectra were acquired from leaves during flowering and machine learning algorithms applied allowed sex classification, which was best using a radial basis function (RBF) network. Eight industrial hemp (low THC) cultivars were field grown on fertilized and control soil. Reflectance spectra were acquired from leaves at early development when the plants of all cultivars had developed between four and six leaf pairs and in three cases only flower buds were visible (start of flowering). Machine learning algorithms were applied, allowing sex classification, differentiation of cultivars and fertilizer regime, again with best results for RBF networks. Differentiating nutrient status and varietal identity is feasible with high prediction accuracy. Sex classification was error‐free at flowering but less accurate (between 60% and 87%) when using spectra from leaves at early growth stages. This was influenced by both cultivar and soil conditions, reflecting developmental differences between cultivars related to nutritional status. Hyperspectral measurement combined with machine learning algorithms is valuable for non‐invasive assessment of *C. sativa* cultivar and sex. This approach can potentially improve regulatory security and productivity of cannabis farming.

## Graphical abstract

We have investigated the possibility of utilizing the leaf spectral phenotype of nine distinct cultivars of *Cannabis sativa* L. for several differentiation tasks by means of machine learning algorithms. Our approach was proven valuable for the non‐invasive assessment of the cultivar and nutritional status as well as for sex prediction very early during plant development under field conditions. We believe that this approach will improve the regulatory security and productivity across all cannabis production systems and discuss the possible monitoring of such traits in other crops.

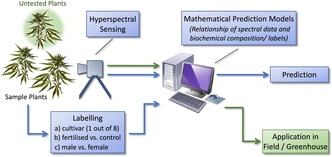

## 1 INTRODUCTION

Industrial hemp (*Cannabis sativa* L.) is a versatile crop which is increasingly used in a broad range of applications and products, including food, health care (Burton et al., 2022; Fike, 2016; Rupasinghe et al., 2020; Schluttenhofer & Yuan, 2017; Williams, 2019) fiber for construction and packaging industries (Awwad et al., 2010; Deshmukh, 2022; Saravanakumar et al., 2021), cosmetics (Vogl et al., 2004), biofuels and phytoremediation (Das et al., 2017; Parvez et al., 2021; Rheay et al., 2021), high‐performance textiles (Musio et al., 2018), and natural insecticides (Benelli et al., 2018). This multifaceted use of almost all parts of the plant has driven improvements in many applications and cultivation practice and increased economic interest in industrial hemp as a viable sustainable crop worldwide (Moscariello et al., 2021; Rehman et al., 2021; Williams, 2019; Wimalasiri et al., 2021).

There have been intensive debates on hemp taxonomy during recent decades, interestingly notwithstanding its rather limited genetic research. However, despite pronounced morphological and phytochemical differences it is common consent that all hemp is *C. sativa* L. (Fike, 2016; Schilling et al., 2020; Williams, 2019). Among *C. sativa* L. a high Δ9‐tetrahydrocannabinol (Δ9‐THC)/low cannabidiol (CBD) and a low Δ9‐THC/high CBD subspecies are recognized with both domesticated and ruderal varieties (Small, 2015, 2017). This diversity is likely related to two linked gene clusters of functional and non‐functional genes, which encode different versions of the two main enzymes involved in either Δ9‐THC or CBD synthesis. In addition, these genes are interspersed with a multitude of mobile genetic elements, making sequencing more challenging and genetic rearrangements in other, not yet sequenced, cultivars more likely (Grassa et al., 2021; Lynch et al., 2016; Schilling et al., 2020). High Δ9‐THC levels characterize medicinal hemp while industrial hemp is defined by high CBD and low Δ9‐THC (<0.1%–1%) levels, depending on the country and state (Duppong, 2009; Schluttenhofer & Yuan, 2017). Today, industrial hemp production and research are allowed in parts of Australia and other countries such as Canada, USA, and in Europe under strict licensing conditions (Schluttenhofer & Yuan, 2017), and production requirements are gaining increasing importance (https://www.agrifutures.com.au/farm‐diversity/industrial‐hemp/, Ellison, 2021; Vogel, 2017). As licensing for farming and research as well as certification of produced goods rely on low Δ9‐THC levels and thus the cultivar used, reliable means of ensuring cultivar identity are mandatory. Current methodologies for subspecies or cultivar assessment rely on morphology traits, which can be unreliable due to the phenotypic plasticity of cannabis in response to its environment (Bernstein et al., 2019; De Meijer & Keizer, 1996) as well as on the biochemical quantification of cannabinoid contents in mature floral material (Borroto Fernandez et al., 2020; ElSohly et al., 2017). For nonflowering material or tissue devoid of cannabinoids, accurate prediction of the chemical phenotype (chemotype; Campbell et al., 2019) and of other morphological, flowering, and biomass quality traits (Faux et al., 2016; Onofri & Mandolino, 2017; Petit et al., 2020) is difficult without costly and time‐consuming downstream analyses. For this reason, progress in more robust analytical assays for hemp, particularly non‐invasive approaches for cultivar and chemotype assessment has been slow.

Many factors, including sowing date in relation to latitude, temperature, available moisture throughout the growing season, varieties, and soil fertility influence hemp growth, seed maturation, and quality (Bennett et al., 2006; Fike, 2016; Irakli et al., 2019; Kostuik & Williams, 2019). Hemp plants are typically dioecious, with tall and thin male plants which die soon after release of pollen and leafy shorter female plants surviving through to seed maturity (Amaducci et al., 1998; Faux et al., 2013; Razumova et al., 2016). Thus, when *C. sativa* L. is grown for seed, fiber, or medicine, sex is an important trait for production, and much effort has been given to understanding its control and in developing lines that are better suited to the desired end use (Hall et al., 2012; Moliterni et al., 2004; Sarkar et al., 2017). Despite genetic control by a pair of heteromorphic sex chromosomes (Faux et al., 2013; Moliterni et al., 2004; Razumova et al., 2016), high plasticity has been observed for the sexual phenotype in cannabis including epigenetic, transcriptional, post‐transcriptional, and hormonal factors controlling sex determination under certain environmental conditions (Galoch, 1978; Hall et al., 2012; Mohan Ram & Sett, 1982; Nelson, 1944). Therefore, cultivation conditions and management practices for medicinal hemp are typically optimized in controlled environments (Jin et al., 2019), which is not possible in the field. Recent approaches for sex determination in cannabis rely on the analysis of genetic markers nearly exclusively (Faux et al., 2016; Onofri & Mandolino, 2017; Sarkar et al., 2017), and the development of methods for plant hormone‐induced feminization is still in its infancy (Flajšman et al., 2021). As this is impractical in real world agriculture, other management practices including the quick and easy assessment of the sex of cannabis plants, preferably prior to planting or very early during development, are required.

Innovative developments for the analysis of biological samples make use of hyperspectral signatures which can be mapped against biochemical compositions non‐destructively, and which can be used to assess plant vitality, stress parameters, nutrition status, and diseases (Backhaus & Seiffert, 2013; Manley, 2014; Wang et al., 2016). Typically, an acquired hyperspectral signature is collectively modulated by the complete biochemical composition of the measured sample. A dedicated mathematical model can then be used to derive specific information from a hyperspectral measurement in the biochemical context of the underlying application. Notably, the discrimination between varieties of various plant species, including tobacco (Seiffert et al., 2010), grapevine (Diago et al., 2013; Gutiérrez et al., 2018), cotton, rice, sugar cane, and chillies (Rao, 2008) from spectral signatures of leaves, have been reported earlier. Recently, the applicability of such approaches has also been reported for cannabis. Sanchez et al. (2020) described the differentiation between cannabis, CBD‐rich plants and industrial hemp based on Raman spectroscopy combined with partial least square discriminant analysis. Similarly, Pereira et al. (2020) showed the potential of near infrared hyperspectral imaging to define *C. sativa* L types., also with a limited number of four indicative spectral bands. Focusing on industrial hemp, Lu et al. (2021) have applied a benchtop hyperspectral imaging system in the spectral range of 400–1000 nm combined with machine learning to differentiate cultivars, growth stages, flowers, and leaves. Notably, prediction of the sexual phenotype of plants from hyperspectral signatures has not been reported yet.

Therefore, we have investigated the possibility of utilizing the leaf spectral phenotype of nine distinct cultivars of industrial hemp for several differentiation tasks by means of machine learning algorithms. Our approach was proven valuable for the non‐invasive assessment of the cultivar and nutritional status as well as for sex prediction very early during plant development under field conditions. We believe that this approach will improve the regulatory security and productivity across all cannabis production systems and discuss the possible monitoring of such traits in other crops.

## 2 MATERIALS AND METHODS

### 2.1 Plant materials and growth

The field site, University of Adelaide, Waite Campus, South Australia, Australia (34°57′58” S 138°38′1″ E) was prepared in December 2019 and January 2020. The site has not been used to grow *C. sativa* L. before and was historically used for experimental trials including wheat, chickpea, barley, sorghum, and maize in typical crop rotation. Following discing, a one‐time application of commercially produced compost (Peat's Soils) was made to an area of roughly one third of the field site (Figure S1). This allowed for cultivating plants in the field under two soil conditions, namely unfertilized (control) and fertilized.

Nine commercial cultivars of *C. sativa* L. were used in this study (Table 1). The cultivar Ferimon 12 was sown into 52‐cell trays with 75% hydrated coco peat (Coir Pith Blocks 4.5 kg; Galuku 330 Exports India Pvt. Ltd.) and 25% coarse sand (Builders 331 Sand). Seedlings were cultivated on trestle tables under a shade cloth at the northern end of the field site for 3 weeks then transplanted into the field on fertilized soil (09/02/2020). After 1 week in the field (17/02/2020, 4 weeks after sowing) Ferimon 12 plants started flowering and reflectance spectra were acquired from the three youngest fully expanded leaves (one spectrum each), from five male and five female plants (30 spectra in total). This dataset was only used for the initial evaluation of the applicability of hyperspectral imaging for sex determination in *C. sativa*, as it varied largely from the other dataset in terms of age and growth conditions of the plants.

**TABLE 1:** List of all dioecious *Cannabis sativa* L. cultivars evaluated.

Cultivar	Source^a^	Origin	Sowing
Puma	HempCorp	China	Field
HAN FN‐Q	HempCorp	China	Field
Si‐1	HempCorp	China	Field
HAN NE	HempCorp	China	Field
Bama	HempCorp	China	Field
HAN COLD	HempCorp	China	Field
HAN FN‐H	HempCorp	China	Field
Yuma	HempCorp	China	Field
Ferimon 12	Cathy Bryant, Neerim, Victoria	France	52‐cell trays under shade cloth

^a^ The Hemp Corporation Pty Ltd (HempCorp).

The cultivars Yuma, HAN FN‐H, HAN COLD, Bama, HAN NE, Si‐1, HAN FN‐Q, and Puma were directly sown into the field site on both unfertilized (control) and fertilized soil (Figure S1). Seven weeks post‐sowing, reflectance spectra were acquired by sampling the three youngest fully expanded leaves (one spectrum each) from 15 plants per cultivar from each soil treatment (90 spectra per cultivar and 720 spectra in total). This was when the plants of all cultivars had developed between four and six leaf pairs. The measured individual plants were labeled (from 1 to 15) and the sexual phenotype was assessed during the period of 7 weeks (17/02/2020) to 16 weeks (20/04/2020) after sowing.

### 2.2 Hyperspectral measurement

Hyperspectral data were acquired with an ASD FieldSpec3 Hi‐Res broadband spectroradiometer (Malvern Panalytical) covering the wavelength range from approx. 350 to 2500 nm, with 2151 wavelength bands, and a measurement spot with a diameter of ~2 cm. Measurements were conducted on a bright day between 9 am and noon. To avoid environmental illumination, a leaf clip was combined with the plant probe. The instrument was calibrated against a circular white target pad (diameter 5 cm, from 15/10/2015, ID SG 3151, Rep. # T15102208, R% 99, SphereOptics GmbH), and baseline corrected using dark current by closing the internal shutter. This calibration procedure was done several times during the whole campaign, to ensure stable measurement results.

### 2.3 Data analysis

**FIGURE 1:**
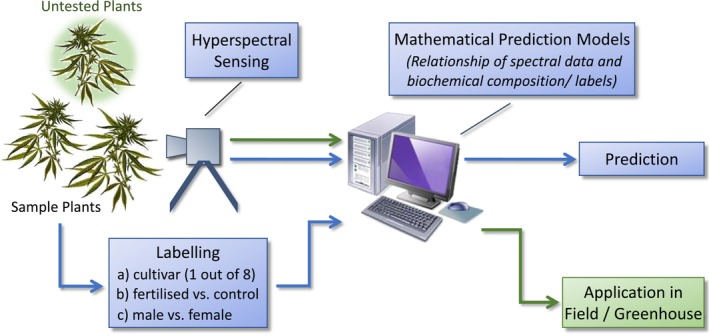
Flowchart of the mathematical modeling process. The acquired hyperspectral signatures are used as input data into the mathematical model. Corresponding reference data are used as output data. According to the three tasks described above, there are three sets of labels used here forming three separate models. Following the structure of the label information, the implemented mathematical model is an 8‐class classifier for task (a) and a binary classifier for task (b) and (c), respectively. Following the general approach of machine learning, the discrepancy (calculated via a standard mathematical error function) between actual and desired output of the model (prediction value) is being minimized during the modeling process. The lower path of the workflow (blue arrows) is not required in productive operation once the mathematical model is sufficiently trained and subsequently used to process all incoming hyperspectral signatures of new (untested) plants based on the learned relationship between input and output data. These yields downstream applications in the field and greenhouse (green arrows).

After acquiring data, further analysis is needed to get meaningful and interpretable results, since the raw spectral fingerprint is non‐specifically modulated by the entire biochemical composition above the limit of detection. The measurements are already available as calibrated reflectance spectra between one and zero, since we already calibrated raw data during the measurements via a white target and the dark current. Various datasets were created in order to test the application of hyperspectral sensing for different tasks under the wide field of industrial hemp (see Table 2). Mathematical models are needed in order to generate human interpretable results from the high‐dimensional hyperspectral datasets. Since the relation between hyperspectral input data and output labels are not known analytically, dedicated mathematical prediction models are generated by a data‐driven approach using machine learning methods. Furthermore, by adding a suitable validation scheme we also obtain comprehensible performance values in the form of a correct classification rate which is a measure for the accuracy value of the classifier for the respective task. The entire process is illustrated in Figure 1.

**TABLE 2:** Overview of all datasets.

Dataset	Measurements per plant	Number of plants	Number of data points	Number of classes
Task (a): cultivars	3	15	45	8
Task (b): soil types	3	240	720	2
Task (c): sex	3	10	30	2

The acquired hyperspectral data are pre‐processed by a vector L2‐normalization before the machine learning applied directly after different task‐specific datasets have been formed from the data. Since there has been little research on hyperspectral data and industrial hemp so far, the machine learning was optimized based on the model selection level. The approach included various machine learning algorithms with different architectures (see Table 3 for further details). In order to test conceptually distinct approaches, we have included fundamentally different architectures. On the one hand, these are linear models (partial least squares [PLS]) versus non‐linear models (multilayer perceptron [MLP], radial basis function network [RBF]). Regarding the (non‐linear) representation of the learned information, we have included hyperplane‐based (MLP) and prototype‐based (RBF) neural architectures. Within the latter class of architectures, we have used different Gaussian kernels, combined with both classical Euclidean metrics and Pearson correlation metrics, as well as divergence as a metric. In the case of divergences, we have focused on Kullback–Leibler divergences as a special case of the more general Gamma divergences with convergence of 𝛾 to zero (Knauer et al., 2015). Training parameters for PLS and MLP models have been kept to the standard values of the MATLAB (The MathWorks, Inc) Deep Learning Toolbox and the Statistics and Machine Learning Toolbox. The RBF network and its training was implemented manually according to Kingma and Ba (2014) with the parameters detailed in Table 3.

**TABLE 3:** Machine learning models used, their architecture and parametrization. Further minor training parameters for PLS1 and MLP have been kept to the standard values of the MATLAB Deep Learning Toolbox and the Statistics and Machine Learning Toolbox. The radial basis function (RBF) network and it's training was implemented manually using Adam learning method (Kingma & Ba, 2014) with the parameters detailed in the table.

Types of models	Architecture	Hyperparameter	Training parameters
Partial least squares (PLS)	PLS1 for categorical vectorial output data, with deflation of input matrix; output/target vector unchanged	5, 20, 50 components	Standard MATLAB values
Multilayer perceptron	1 hidden layer (fixed size, variable width of layers)	10, 25, 50 neurons	Learning method: Scaled conjugate gradient backpropagation (*trainscg*); Epochs: 500; Max validation fails: 20; Hidden layer transfer function: Hyperbolic tangent sigmoid transfer; Output layer transfer function: Linear
	2 hidden layers (fixed size, pyramid‐shaped layers)	First layer: 50 neurons Second layer: 30 neurons	Standard MATLAB values
RBF network	Gaussian kernel + Euclidean metric	5, 10, 15, 40 prototypes	Learning method: Adam Step size: 0.01; Exponential decay rate for 1st and 2nd momentum estimates: 0.6 and 0.999; Stabilization ϵ: 1^e‐8^; Epochs: 400; Max validation fails: 20
	Gaussian kernel + weighted Euclidean metric		
	Gaussian kernel + Pearson correlation metric		
	Gaussian kernel + Kullback–Leibler divergence		

In the end, we used the mean correct classification rate as the typical measure for accuracy to assess the performance of all individual model candidates. Particularly in the case of the multiple classifiers (Task (a): cultivars), we used the respective confusion matrices to uncover the specific misclassifications between individual classes and to use them as a further selection criterion for the previously generated candidate models. In order to test all models under realistic conditions, in addition to the standard statistical n‐fold cross‐validation, a *complete* leave‐one‐out validation scheme was followed that leaves one complete plant out of the machine learning training. In this context, the term *complete* means, that this procedure was repeated until each plant in the dataset acts as a validation plant. All validation cycles are averaged based on their individual performance measures and reported as mean and standard deviation values accordingly (Tables 4, 5, 6).

**TABLE 4:** Confusion matrix for the differentiation between male and female plants of cultivar Ferimon 12. Correct classification rate by individual measurement applying leave‐one‐out validation is shown. Shown in brackets are the values for correct classification rate by entire plant applying leave‐one‐out validation and majority voting, that is, the class that wins the majority of all decisions is considered the winning class. The mean and standard deviation were 1 and 0 for both mathematical models. All spectra of male and female plants were correctly assigned. The best‐performing model was an radial basis function using Euclidean metric and 10 prototypes. Respective F1, precision, and recall values are shown in Table S4.

	True class	
	Male	Female
Predicted class		
Male	15 (5)	0 (0)
Female	0 (0)	15 (5)

In a confusion matrix, the diagonal line is typically highlighted as ideal classification. If all data would be correctly assigned only these cells in the table would contain data. In a more realistic scenario, as in this study, there are misclassifications to be found in non‐diagonal cells (In grey).

**TABLE 5:** Confusion matrix for the differentiation between cultivars. Correct classification rate by individual measurement applying leave‐one‐out validation is shown. The mean and standard deviation were 0.997 and 0.03. Shown in brackets are the values for correct classification rate by entire plant applying leave‐one‐out validation and majority voting. Here, the mean and standard deviation were 1 and 0. All spectra of all cultivars were correctly assigned, except one spectrum of cultivar Yuma. The best‐performing model was an radial basis function using weighted Euclidean metric and 40 prototypes.

	True class							
	Yuma	HAN FN‐H	HAN COLD	Bama	HAN NE	Si‐1	HAN FN‐Q	Puma
Predicted class								
Yuma	44 (15)	0 (0)	0 (0)	0 (0)	0 (0)	0 (0)	0 (0)	0 (0)
HAN FN‐H	0 (0)	45 (15)	0 (0)	0 (0)	0 (0)	0 (0)	0 (0)	0 (0)
HAN COLD	0 (0)	0 (0)	45 (15)	0 (0)	0 (0)	0 (0)	0 (0)	0 (0)
Bama	0 (0)	0 (0)	0 (0)	45 (15)	0 (0)	0 (0)	0 (0)	0 (0)
HAN NE	1 (0)	0 (0)	0 (0)	0 (0)	45 (15)	0 (0)	0 (0)	0 (0)
Si‐1	0 (0)	0 (0)	0 (0)	0 (0)	0 (0)	45 (15)	0 (0)	0 (0)
HAN FN‐Q	0 (0)	0 (0)	0 (0)	0 (0)	0 (0)	0 (0)	45 (15)	0 (0)
Puma	0 (0)	0 (0)	0 (0)	0 (0)	0 (0)	0 (0)	0 (0)	45 (15)

In a confusion matrix, the diagonal line is typically highlighted as ideal classification. If all data would be correctly assigned only these cells in the table would contain data. In a more realistic scenario, as in this study, there are misclassifications to be found in non‐diagonal cells (In grey).

**TABLE 6:** Confusion matrix for the differentiation between soil types. Correct classification rate by individual measurement applying leave‐one‐out validation is shown. The mean and standard deviation were 0.988 and 0.07. Shown in brackets are the values for correct classification rate by entire plant applying leave‐one‐out validation and majority voting. Here, the mean and standard deviation were 0.996 and 0.03. Most spectra of the two soil types were correctly assigned with five and four spectra wrongly classified for the control and fertilized group, respectively. The best‐performing model was an radial basis function using Euclidean metric and 40 prototypes. Respective F1, precision, and recall values are shown in Table S4.

	True class	
	Control	Fertilized
Predicted class		
Control	355 (120)	4 (1)
Fertilized	5 (0)	356 (119)

In a confusion matrix, the diagonal line is typically highlighted as ideal classification. If all data would be correctly assigned only these cells in the table would contain data. In a more realistic scenario, as in this study, there are misclassifications to be found in non‐diagonal cells (In grey).

The approach described here, including validation and model selection, has been implemented in the MATLAB (The MathWorks, Inc) software environment.

## 3 RESULTS

### 3.1 Proof of concept: Sex classification was highly accurate from leaf spectra taken at early flowering stage

**FIGURE 2:**
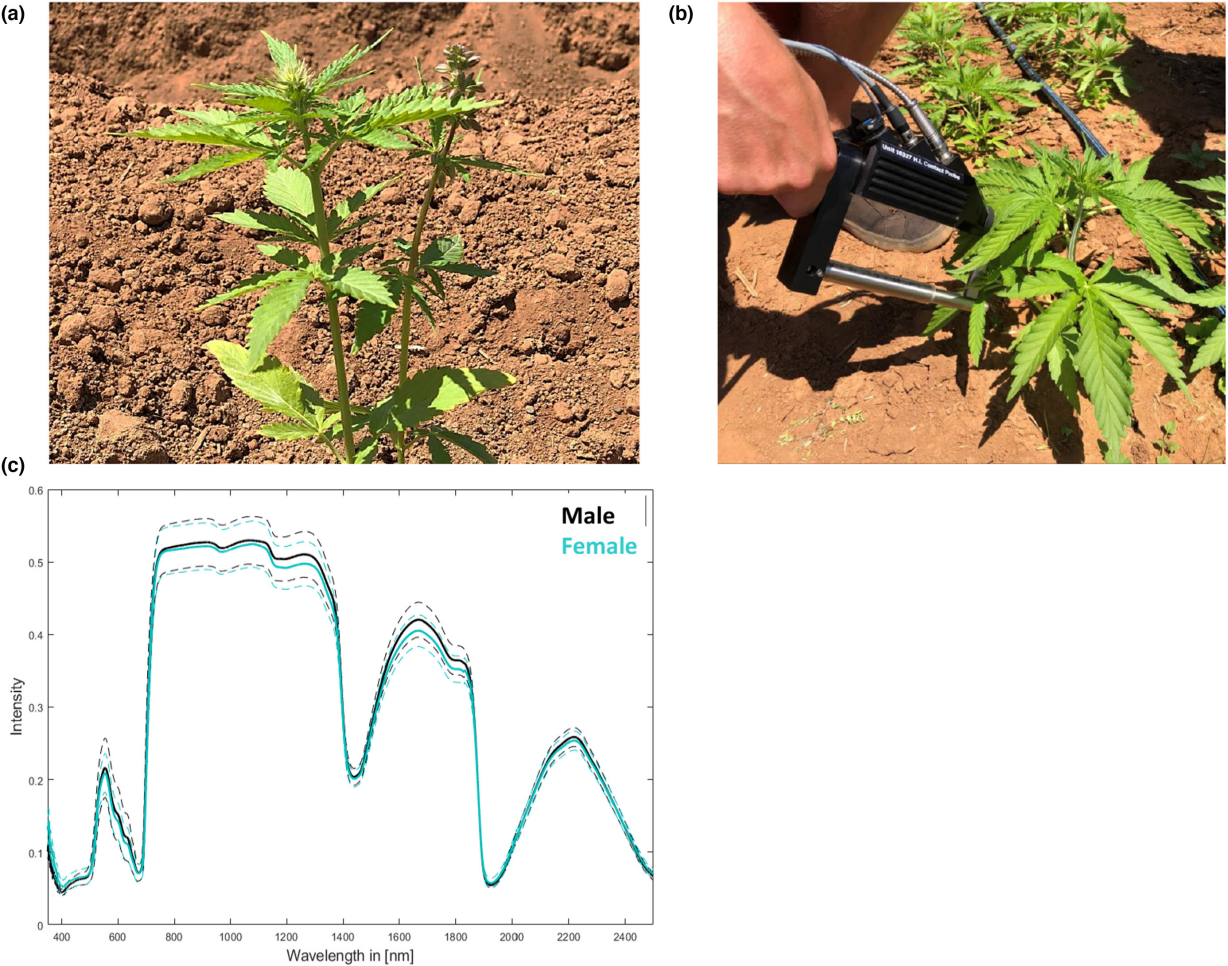
Hyperspectral measurement of flowering dioecious *Cannabis sativa* L. plants from cultivar Ferimon 12. (a) A female (left) and a male (right) plant of the cultivar Ferimon 12 at the time point of measurement (17/02/2020, 4 weeks after sowing and 1 week after planting into the field). (b) The actual measurement of a leaf with the field portable full range spectroradiometer. To avoid environmental illuminations, a leaf clip was combined with the plant probe. (c) The mean reflectance spectra acquired for the cultivar Ferimon 12 from three leaves per plant from five male and five female plants, resulting in 15 spectra each. Dotted lines indicate the variance range.

To confirm the applicability of our chosen approach, we first investigated flowering male and female *C. sativa* L. plants of the cultivar Ferimon 12 (Figure 2). In contrast to all other plants used in this study, plants of this cultivar were cultivated in pots for 3 weeks before being transplanted into fertilized soil in the field. These plants began to flower very early, namely 4 weeks after sowing (Figure 2a). Using a field portable full range spectroradiometer we acquired reflectance spectra from three leaves per plant from five male and five female plants, resulting in 15 spectra each (Figure 2b). By visual inspection of the mean reflectance spectra differences between the sex specific profiles could already be observed, which seemed to be mainly related to intensity differences in distinct wavelength ranges (Figure 2c). These data were used for calculating classification models for the differentiation between male and female plants of cultivar Ferimon 12. Correct classification rates, that is, the number of correct decisions of the classifier divided by the total number of data points, are shown as confusion matrix, that is, the distribution of decisions of the classifier over all classes, both for the individual measurements and for the entire plants (Table 4). All spectra of male and female plants were correctly assigned. This result confirmed that the biochemical information on the leaf surface of flowering *C. sativa* L. plants, which is directly reflected in the spectral profiles, is distinct enough to differentiate between male and female plants. We postulated that this physiological diversity may likely develop early during the growth phase, and thus enable the early classification of sex of cannabis plants. Such physiological variations possibly also allow for the differentiation between developmental stages in general and between cultivars or varieties.

### 3.2 Discrimination of varieties is possible with high accuracy at early plant development

**FIGURE 3:**
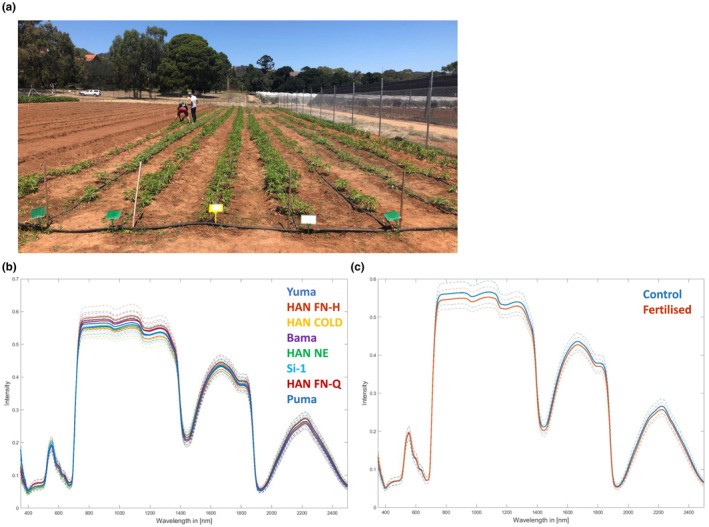
Hyperspectral measurements of dioecious *Cannabis sativa* L. plants before flowering. (a) The field setting at the measurement day (17/02/2020). *C. sativa* L. cultivars from left to right are Yuma, HAN FN‐H, HAN COLD, Bama, HAN NE, Si‐1, HAN FN‐Q, and Puma. Spectra were acquired from three leaves per plant from 15 plants per cultivar from two soil conditions (fertilized and control), resulting in 90 spectra per cultivar and 720 spectra in total. (b) The mean reflectance spectra of the eight different cultivars across both soil conditions, and (c) the mean reflectance spectra of the two soil conditions across all cultivars. Dotted lines indicate the variance range.

Next, we investigated plants of eight dioecious *C. sativa* L. cultivars, namely Yuma, HAN FN‐H, HAN COLD, Bama, HAN NE, Si‐1, HAN FN‐Q, and Puma, during early plant development. Plants were cultivated in the field under two soil conditions, namely unfertilized (control) and fertilized (Figure S1). Reflectance spectra were acquired 7 weeks after sowing (Figure 3a) from three leaves per plant from 15 plants per cultivar from each soil condition, resulting in 90 spectra per cultivar, and 720 spectra in total. Plants were further cultivated until maturity and sex of the individual plants, which had been labeled from one to 15 at the measurement date, was assigned during a period of seven to 16 weeks after sowing. The resulting dataset was utilized for three separate analyses: to calculate classification models for (i) the differentiation between the eight cultivars, (ii) the differentiation between plants grown on either fertilized or control soil, and (iii) the differentiation between male and female plants.

**FIGURE 4:**
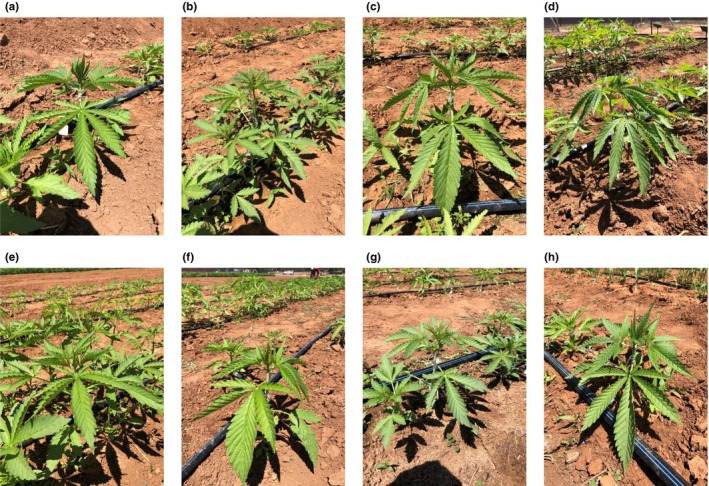
Images of all dioecious *Cannabis sativa* L. cultivars evaluated at the measurement day (17/02/2020). (a) Yuma, (b) HAN FN‐H, (c) HAN COLD, (d) Bama, (e) HAN NE, (f) Si‐1, (g) HAN FN‐Q, and (h) Puma.

Between cultivars the mean reflectance spectra indicated differences between the specific profiles, mostly related to intensity differences in distinct wavelength ranges (Figure 3b). Additionally, minor developmental and phenotypic differences between the cultivars could be observed visually (Figure 4). We noticed, regardless of the soil condition, that plants of the cultivars HAN FN‐H (Figure 4b) and HAN FN‐Q (Figure 4g) were smaller (e.g., height and leaf size) and were less green in color, being more blue/black instead. All other cultivars appeared phenotypically very similar at the measurement day. However, from the reflectance spectra, discrimination of the cultivars was possible with high accuracy at early plant development. Correct classification rates are shown as a confusion matrix, both for the individual measurements and for the entire plants (Table 5). All spectra of all cultivars were correctly assigned, except one spectrum of cultivar Yuma, resulting in a mean value of 0.997 with a standard deviation of 0.03 for the cultivar prediction based on individual measurements. These results suggest that the physiological variation between the eight cultivars tested in our study is high enough to enable the prediction of *C. sativa* L. cultivars early during plant development and regardless of the soil condition.

### 3.3 Soil fertility strongly influences plant physiology and thus spectral profiles

Figure 3c shows the mean reflectance spectra of the two soil conditions across all cultivars highlighting differences in intensity of several wavelength regions. Accordingly, the mathematical models developed allowed for the highly accurate discrimination between the two soil conditions (Table 6). Most spectra relating to the two soil conditions were correctly assigned with only five and four spectra wrongly classified for the control and fertilized group, respectively. The resulting mean and standard deviation were 0.988 and 0.07. For the entire plant, one plant was not correctly assigned from the “fertilized” dataset leading to a mean and standard deviation of 0.996 and 0.03. It can be assumed that the differences in soil fertility have influenced plant growth and development, which is reflected in changed spectral profiles. The changes observed in spectral profiles differed between the cultivars, were noticed to be strongest for Yuma, HAN FN‐H, and Bama, and were lowest for HAN COLD and Si‐1 (Figure S2). However, all spectra of the two soil types were correctly assigned when we calculated the correct classification rates for the cultivars individually (Table S1). These data indicate that plant development has changed in a nutrition‐ and cultivar‐dependent manner, which may likely also affect flower development and maturation and thus the development of mathematical prediction models related to this task.

### 3.4 Prediction of sexual phenotype is possible with good accuracy at early plant development

**FIGURE 5:**
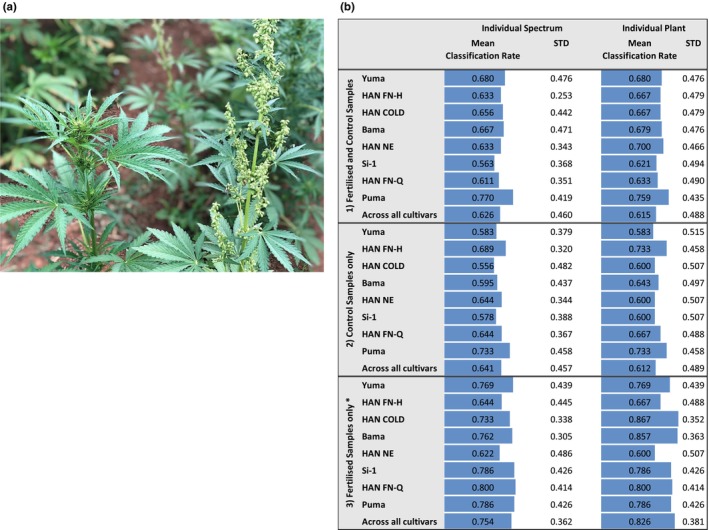
Early prediction of sex of dioecious *Cannabis sativa* L. plants. (a) A photographic image of a female (left) and a male (right) plant of the cultivar HAN FN‐H at the time point of sex annotation (05/03/2020, 9 weeks after sowing into the field). (b) The mean accuracies for the prediction of sex from reflectance spectra measured from leaves before flowering (17/02/2020). Compared are the mean classification rates by individual measurement and by entire plant for three datasets: (1) containing all spectra acquired from each cultivar (fertilized and control conditions, 15 plants each with three leaves per plant measured; 90 spectra per cultivar in total), (2) containing all spectra acquired from each cultivar grown on control conditions (15 plants each with three leaves per plant measured; 45 spectra per cultivar in total), and (3) containing all spectra acquired from each cultivar grown on fertilized conditions (15 plants each with three leaves per plant measured; 45 spectra per cultivar in total). Significant improvement of classification rates for plants grown under fertilized conditions is indicated by *; paired *t*‐test, *p* = .0015 (individual spectra) and *p* = .0044 (entire plant) when compared with dataset (1) as well as *p* = .0005 (individual spectra) and *p* = .0006 (entire plant) when compared with dataset (2).

In our experimental setup, reflectance spectra were acquired from leaves of all cultivars on the same day (17/02/2020, 7 weeks after sowing). Assessment of sex of individual plants was then performed for the measured and labeled (number one to number 15) plants when flowering started, between 17/02/2020 and 10/04/2020. An indicative photographic image of a female (left) and a male (right) plant of the cultivar HAN FN‐H at the time point of sex annotation is shown in Figure 5a. We noticed generally higher numbers of female plants, except for cultivar Si‐1 under control conditions and cultivar HAN FN‐Q under fertilized conditions (Table S2). Some plants died in the course of the experiment: which was the case for the cultivars Yuma (three plants) and Bama (one plant) under control conditions, as well as for the cultivars Yuma (two plants), Bama, Si‐1, and Puma (one plant each) under fertilized conditions. The resulting dataset (reflectance spectra plus sex annotation) was then utilized for calculating mathematical models for the early prediction of male and female plants from the leaf spectral profiles. In Figure 5b, the mean classification rates by individual measurement and by entire plant are shown utilizing spectra either of the individual cultivars or spectra across all cultivars for three model generation datasets: (1) containing all spectra acquired from each cultivar (fertilized and control conditions), (2) containing all spectra acquired from each cultivar grown in control soil conditions, and (3) containing all spectra acquired from each cultivar grown in fertilized soil conditions. The resulting mean classification rates are shown for predictions of the different cultivars and across all cultivars. Within dataset (1) best results were obtained for the sex prediction of the cultivar Puma with mean classification rates (standard deviation) of 0.770 (0.419) for the individual spectra and 0.759 (0.435) for the entire plants (Figure 5b, top box). Mean classification rates for all other cultivars were observed to be very similar. Within dataset (2) we obtained best results for the sex prediction of the cultivars HAN FN‐H and Puma with mean classification rates (standard deviation) of 0.689 (0.320) and 0.733 (0.458) for the individual spectra, and 0.733 (0.458) and 0.733 (0.458) for the entire plants, respectively (Figure 5b, middle box). Mean classification rates for all other cultivars were observed to be very similar again. Significant improvement of classification rates for all cultivars, except HAN NE and HAN FN‐H, was observed for plants grown under fertilized conditions (dataset 3) (Figure 5b, bottom box). For predictions by individual spectrum mean classification rates between 0.622 (HAN NE) and 0.800 (HAN FN‐Q) were reached, with *p* = .0046 and *p* = .0038 when compared across all cultivars within dataset (1) and (2), respectively. Similarly, for predictions based on entire plants, mean classification rates between 0.600 (HAN NE) and 0.867 (HAN COLD) were reached, with *p* = .0098 and *p* = .0039 when compared across all cultivars within dataset (1) and (2), respectively.

Generally, high standard deviations were noted for all sex prediction models, which we could link to wrong assignment of entire plants (Table S3). To aid further interpretation a table with F1, precision, and recall values for all classification tasks is included (Table S4). When we evaluated the classification rates of individual plants, obtained for the three datasets, a considerable number of false predictions was observed. Most plants showed either correct assignment of all three spectra (value 1, orange) or of two out of three spectra (0.667, light orange), while for several plants false assignment of all three spectra (0, blue) or of two out of three spectra (0.333, light blue) was detected. The prediction accuracy for the individual plants increased significantly (*p* = .0046 and *p* = .0038 when compared to dataset 1 and 2, respectively) for plants grown under fertilized conditions (Table S3, bottom box), which is also reflected by the enhanced mean classification rates of the individual cultivars and across all cultivars (Figure 5b, bottom box).

When we evaluated the confusion matrices for the differentiation between sex per cultivar (Table 7) it was apparent that predictions of males failed more frequently than predictions of females. This observation was generally most pronounced for the datasets (1) and (2) and was less strong for the dataset (3) containing only data from fertilized conditions. Across all cultivars for dataset (1) 142 female and 0 male, for dataset (2) 70 female and 1 male, as well as for dataset (3) 72 female and 23 male plants were correctly classified, whereas 0 female and 89 male (dataset 1), 0 female and 45 male (dataset 2), as well as 0 female and 20 male plants (dataset 3) were falsely predicted (Table 7, bottom two rows). Once again, these results strongly indicate a better prediction potential for plants which were cultivated under fertilized conditions. On the other hand, prediction accuracy clearly depends on a sufficient and balanced number of available sample data, which was not always the case for our sex data (Table S2). This assumption is further supported by data obtained for cultivars with either higher number of males (HAN FN‐Q, fertilized conditions (dataset 3), 12 male (M) and 3 female (F) plants) or similar numbers of males and females in the dataset, thus producing better classification rates for male plants also under control conditions (dataset 2); namely Yuma (5M, 7F), HAN FN‐H (6M, 9F), Si‐1 (8M, 7F), and HAN FN‐Q (6M, 9F). In contrast, low male prediction accuracies were observed under control conditions for Puma, a cultivar with a high number of females (4M, 11F), as well as for Yuma (3M, 10F), Si‐1 (3M, 11F) and Puma (3M, 11F) under fertilized conditions.

**TABLE 7:** Confusion matrices for the differentiation between sex per cultivar. Correct classification rates by individual measurement applying leave‐one‐out validation from using either spectra of the individual cultivars or spectra across all cultivars are compared for three datasets: (1) containing all spectra acquired from each cultivar (fertilized and control conditions, 15 plants each with three leaves per plant measured; 90 spectra per cultivar in total), (2) containing all spectra acquired from each cultivar grown on control conditions (15 plants each with three leaves per plant measured; 45 spectra per cultivar in total), and (3) containing all spectra acquired from each cultivar grown on fertilized conditions (15 plants each with three leaves per plant measured; 45 spectra per cultivar in total). Shown in brackets are the values for correct classification rate by entire plant applying leave‐one‐out validation and majority voting. The mean and standard deviation are shown in Figure 5. Significant improvement of classification rates for plants grown under fertilized conditions is indicated by *. The best‐performing models for the individual classifications were (a) an radial basis function (RBF) using Euclidean metric and 5 prototypes, (b) PLS1 with 20 components, (c) MLP with 1 hidden layer and 10 neurons, (d) an RBF using Euclidean metric and 15 prototypes. Respective F1, precision, and recall values are shown in Table S4.

	True class					
	(1) Fertilized + control		(2) Control samples only		(3) Fertilized samples only*	
	Female	Male	Female	Male	Female	Male
Predicted class						
Yuma						
Female	40^(a)^ (17)	24 (8)	10^(b)^ (3)	4 (1)	30^(a)^ (19)	9 (3)
Male	11 (0)	0 (0)	11 (4)	11 (4)	0 (0)	0 (0)
HAN FN‐H						
Female	32^(b)^ (10)	14 (3)	20^(b)^ (7)	7 (2)	18^(b)^ (6)	10 (3)
Male	19 (7)	25 (10)	7 (2)	11 (4)	6 (2)	11 (4)
HAN COLD						
Female	57^(c)^ (20)	28 (10)	23^(c)^ (8)	16 (5)	29^(c)^ (11)	8 (2)
Male	3 (0)	2 (0)	4 (1)	2 (1)	4 (0)	4 (2)
Bama						
Female	56^(a)^ (19)	27 (9)	24^(c)^ (9)	14 (5)	25^(c)^ (9)	5 (1)
Male	1 (0)	0 (0)	3 (0)	1 (0)	5 (1)	7 (3)
HAN NE						
Female	42^(a)^ (15)	24 (7)	24^(a)^ (9)	13 (6)	7^(b)^ (2)	0 (0)
Male	9 (2)	15 (6)	3 (0)	5 (0)	17 (6)	21 (7)
Si‐1						
Female	44^(a)^ (18)	28 (11)	12^(a)^ (4)	10 (3)	33^(d)^ (11)	9 (3)
Male	10 (0)	5 (0)	9 (3)	14 (5)	0 (0)	0 (0)
HAN FN‐Q						
Female	22^(b)^ (6)	21 (5)	17^(b)^ (5)	6 (1)	0^(c)^ (0)	0 (0)
Male	14 (6)	33 (13)	10 (4)	12 (5)	9 (3)	36 (12)
Puma						
Female	66^(c)^ (22)	20 (7)	33^(a)^ (11)	12 (4)	33^(a)^ (11)	9 (3)
Male	0 (0)	1 (0)	0 (0)	0 (0)	0 (0)	0 (0)
Across all						
Female	418^(d)^ (142)	251 (89)	210^(a)^ (70)	125 (45)	203^(a)^ (72)	72 (20)
Male	8 (0)	16 (0)	0 (0)	13 (1)	13 (0)	57 (23)

In a confusion matrix, the diagonal line is typically highlighted as ideal classification. If all data would be correctly assigned only these cells in the table would contain data. In a more realistic scenario, as in this study, there are misclassifications to be found in non‐diagonal cells (In grey).

### 3.5 Flowering time depends on the fertilization status in a cultivar‐specific manner

**FIGURE 6:**
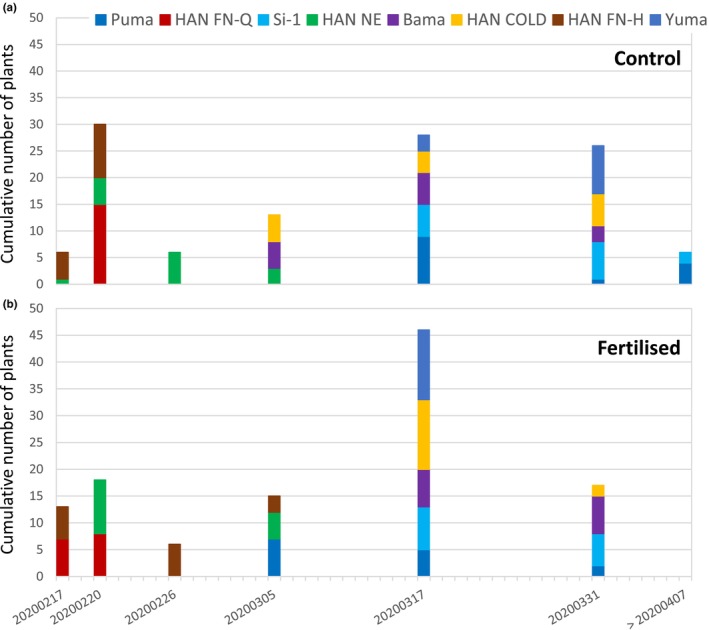
Comparison of flowering time of the eight *Cannabis sativa* L. cultivars. Numbers of plants per cultivar which flowered at a certain date are represented as stacked bars. (a) shows results from plants grown under control conditions and (b) from plants grown under fertilized conditions.

The development of dioecious *C. sativa* L. plants, including flowering, largely depends on environmental factors and differs between varieties (Bennett et al., 2006; Fike, 2016; Kostuik & Williams, 2019). Accordingly, we detected strong genotypic variations for plant development and flowering time in our experiment (Figure 6). Under control conditions the cultivars HAN FN‐Q, HAN NE, and HAN FN‐H flowered early compared to the other cultivars (PUMA, BAMA, HAN COLD, Si‐1, and Yuma, Figure 6a). This effect was compromised for some cultivars by fertilization with compost, resulting in a broader spread (HAN FN‐H) or delay of flowering time (HAN NE, Bama, HAN COLD). In contrast Puma, HAN FN‐Q, Si‐1, and Yuma started flowering earlier when cultivated under fertilized conditions (Figure 6b). Generally, the phenotype of the cultivars HAN FN‐H and HAN FN‐Q was characterized by smaller plants and bluish/black colored leaves throughout plant development (Figure 4b,g; Figure S3).

A correlation of the flowering time with the mean classification rate was observed for several cultivars. The cultivars HAN FN‐Q, HAN NE, and HAN FN‐H flowered early and showed the best mean classification rates under control conditions (dataset 2; Figure 5b, middle box, Figure 6a). Accordingly, for the cultivars HAN FN‐Q, Si‐1, Puma, and Yuma flowering was observed to start earlier when plants were cultivated under fertilized conditions, which were associated with increased mean classification rates for those lines. In contrast, flowering of HAN NE, and HAN FN‐H was delayed under fertilized conditions resulting in similar and even reduced mean classification rates. However, despite showing a delay of flowering the mean classification rates were observed to be increased for the cultivars HAN COLD and Bama under fertilized conditions (dataset 3; Figure 5b, bottom box, Figure 6b).

Together, our results suggest that classification of the sex of *C. sativa* L. plants is possible from leaf reflectance spectra long before flowering and prediction accuracy depends mostly on sufficient size and balance of the training dataset, the nutrition status of the plants, and length of time until flowering, which occurs in a cultivar‐dependent manner.

## 4 DISCUSSION

We have proven the applicability of hyperspectral measurement combined with machine learning algorithms to predict the sex of *C. sativa* L. plants of cultivar Ferimon 12 from leaf reflectance spectra. Clearly, the biochemical information on the leaf surface and in the upper leaf cell layers at the early flowering stage is distinct enough to allow for the generation of mathematical models for the classification of male and female plants (Table 4). This assumption is supported by a study reporting pronounced differences in the content of polyphenols, flavones, and soluble protein, as well as differential activities of peroxidases and catalases, between leaves of male and female *C. sativa* L. plants (Elena et al., 2002). Results from this study matched the early observation of Talley (1934) that carbon to nitrogen ratios (C:N) of the above ground plant material differ between the sexual phenotypes, with higher C‐content in male and higher N‐content in female plants. Among the numerous reported phytocannabinoids, Δ9‐THC is generally considered to be more abundant in female plants, however being most concentrated in trichomes of the female inflorescence (leaves and buds); see ElSohly et al. (2017) and references therein. Also, several plant growth regulators (e.g., gibberellic acid, abscisic acid, and indole acetic acid) have been reported to be involved in the control of flowering and the sexual phenotype in hemp (Galoch, 1978; Hall et al., 2012). However, comprehensive profiling studies comparing male and female metabolic phenotypes are missing. To support the early prediction of sex in cannabis, kinetic studies uncovering the turning point at which the male and female phenotype can be distinguished metabolically would be needed as discussed below.

There is clearly large variability of chemical and morphological phenotypes of *C. sativa* L. (Grassi & McPartland, 2017; Petit et al., 2020; Strzelczyk et al., 2021), which in most cases determine the end use of the cultivar (Fike, 2016; Rehman et al., 2021). In our approach a panel of eight industrial hemp cultivars could be classified with high accuracy from leaf reflectance spectra (Table 5). This is in accordance with the results presented by (Lu et al., 2021) where machine learning based on regularized linear discriminant analysis achieved an accuracy of up to 99.6% in differentiating five industrial hemp cultivars. We assume that this high prediction accuracy mainly relates to distinct chemotypes of the cultivars in our study, which have been selected based on commercial relevance for hemp seed production and from different vendors (Table 1). As the geographical and ecological range of cannabis is unusually broad, substantial differences in the genotypes and thus in the chemotypes can be expected, as reported earlier by Lynch et al. (2016) and Borroto Fernandez et al. (2020) for European hemp varieties. This variability evidently generates a high demand for innovative technologies to cheaply, quickly, and non‐invasively determine cultivar identity in the growing cannabis market, for which the approach presented here proves to be ideally suited. However, despite the increasing scientific evidence for the applicability of spectroscopic approaches to discriminate plant varieties, such as shown for tea (Li & He, 2008), tomato (Xu et al., 2009), eucalyptus (Kumar et al., 2010), tobacco (Seiffert et al., 2010), grapevine (Diago et al., 2013), and hemp (Lu et al., 2021) their industrial applications are still missing (Dos Santos et al., 2013; Lopes & Sousa, 2018). Recently, an automated system combining hyperspectral imaging and machine learning for the classification of grapevine varieties under field conditions has been described (Gutiérrez et al., 2018).

Hemp is an oilseed crop, taking up more nitrogen and potassium, and similar amounts of phosphate as canola (*Brassica napus* L.) and performing well on highly fertile soil. Although it can be cultivated on a wide range of soil types, soil characteristics such as salinity, compaction and high acidity or alkalinity should be avoided (Kostuik & Williams, 2019). As the nutritional status of the plants determines the rate of seed set, good fertilization management is mandatory to maintain hemp yield in the field. In our study, a clear influence of soil management was observed, resulting in pronounced physiological differences between the plants grown on fertilized versus control soil. Leaf reflectance spectra were obviously distinct between the two groups, leading to a very precise classification for all cultivars investigated (Table 3; Table S1). We assume this to be related to different biochemical profiles in all cultivars related to the metabolized compost (better availability of nitrogen) in the plants on fertilized soil. Accordingly, increasing rates of animal manure, nitrogen, and phosphate fertilizers have been reported to enhance stem height and diameter, leaf, and stem weights, as well as the percentage of soluble extract (Laleh et al., 2017; Van der Werf, 1991).

The differences in soil fertility and therefore nutrition status of the plants have also influenced the early classification of sex from leaf reflectance spectra of young *C. sativa* L. plants (Figure 5). Prediction accuracies were highest for plants grown on fertilized soil in a cultivar‐dependent manner. However, improved prediction accuracies were not observed to be directly linked with accelerated plant development and thus altered flowering time (Figure 6; Table 7). Therefore, we concluded a combination of biochemical and morphological factors to be related to these differences, with likely higher impact of the chemotype. *C. sativa* L. is a complex species with overall highly variable morpho‐anatomical features, whereas most studies only concentrate on stem/fiber or trichome type and density characteristics (Raman et al., 2017). A study investigating diversity patterns among a collection of *C. sativa* L. genotypes identified the contents of bark fiber and cannabinoids as the highest discriminating factors (De Meijer & Keizer, 1996). Recently, substantial spatial gradients in secondary metabolite profiles *in planta* were observed which hint at organ and location‐specific regulation of accumulation in *C. sativa* L. (Bernstein et al., 2019), and which possibly vary between different genotypes. In addition, it was shown that *C. sativa* L. glandular trichomes, which are highly enriched on floral organs, alter morphology and metabolite content during flower maturation (Livingston et al., 2020), and similar changes may occur on leaf surfaces. We concluded that growing *C. sativa* L. on fertilized soil may have resulted in increased metabolite content and likely changed metabolite profiles in a cultivar‐specific manner, thus resulting in the differently enhanced prediction accuracies in our study. The observed reduced influence of flowering time on early sex prediction may relate to the time point of data acquisition, which we performed when the plants of all cultivars had developed between four and six leaf pairs. This assumption is supported by a molecular‐morphological study, in which microscopic analysis of male and female apices revealed that their reproductive commitment likely occurs as soon as the leaves of the fourth node emerge (Moliterni et al., 2004).

The most momentous effect on accuracy of early sex prediction was linked to the number of samples in the training datasets for the respective classes (male and female, see Table S2), with slightly higher sample numbers leading to significantly better accuracies. Class imbalance of training data, for example, one class heavily outnumbering the examples in the other class such as commonly happens in biological and medical datasets, has been frequently reported as a factor that negatively influences the performance achieved by existing learning systems (Batista et al., 2004; Vabalas et al., 2019). This situation typically leads to difficulties for the learning system to learn the concept related to the minority class. To overcome this problem the application of over‐sampling methods, for example, Random over‐sampling (Batista et al., 2004; Xiaolong et al., 2019) and the design of robust testing methodologies, for example, nested cross‐validation and train/test split approaches (Musto et al., 2021; Vabalas et al., 2019) have been suggested. Such approaches may also improve future prediction accuracies for the application presented here for cannabis sex prediction.

With respect to the limitations mentioned above, future improvements of the described approach for early sex prediction should include the systematic assessment and evaluation of data from cannabis plants during the course of development, from controlled environments and field trials, including larger numbers of cultivars, particularly medicinal types, and with a clear focus on sufficiently sized and balanced training datasets. In addition, limits for prediction accuracies need to be defined by *C. sativa* L. breeders and farmers, which allow for the economically viable application of spectroscopy‐based approaches for early sex determination in field and greenhouse environments. The methodology described here may also be applicable to other dioecious species of industrial and medical importance, such as date palm, kiwifruit, pistachio, yam, and jojoba, for which identification of the sexual phenotype at the seedling stage is of great importance to breeders and farmers for crop improvement and productivity (Sarkar et al., 2017).

Regarding cannabis, the application of classification approaches based on hyperspectral measurements combined with machine learning algorithms may possibly be extended to the prediction of Δ9‐THC and CBD contents, allowing state of the art non‐destructive analysis of plant chemotypes (Lopes & Sousa, 2018; Manley, 2014; Sanchez et al., 2020; Wang et al., 2016). Also, as shown for the classification of grapevine varieties from seeds (Zhao et al., 2018), the discrimination of cannabis cultivars may be possible even before sowing. For the monitoring and discrimination of species and cultivars in large and heterogenous field trials, airborne‐based approaches may be developed, such as shown earlier for cotton, rice, sugar cane, and chilies (Rao, 2008), and recently for hemp (Pereira et al., 2020). Generally, mathematical prediction models can be developed for a wide range of scales of spectral sensors, and thus may allow the creation of monitoring systems ranging from handheld to airborne devices in the future.

## 5 CONCLUSION

We believe that the application of analytical technologies based on specific spectral signatures combined with computational methodologies will enable the large‐scale assessment of plant varieties, as shown here for cannabis cultivars, and thus improve future control of relevant lines and use in breeding processes, both in terms of speed and costs. In addition, such approaches can help to monitor the nutritional status of plant populations and to identify target plants in dioecious species. As differences in nutritional status obviously influence the ability to identify sexes before flowering, this needs to be taken into account in crop management practices, such as the usage of fertilizers.

## CONFLICT OF INTEREST STATEMENT

The authors have no conflicts of interest to declare. All co‐authors have seen and agree with the contents of the manuscript.

## Supporting information


Appendix S1.

## Data Availability

The data that founded the basis and support the findings of this study are available from the Supporting Information file “SI_Matros_et_al_Hyperspectral_Cannabis_RawData.csv”.
