# Supplementary material for: Non‐invasive assessment of cultivar and sex of Cannabis sativa L. by means of hyperspectral measurement
Source: Plant Environ Interact. 2023 Aug 17;4(5):258–74. doi: 10.1002/pei3.10116 (PMC10564378; doi:10.1002/pei3.10116)
Supplement: Supplementary file 1 — Appendix S1. [file PEI3-4-258-s001.zip › SI_Matros_et_al_Hyperspectral_Cannabis_R2_clean.docx]

## *Plant-Environment Interactions* Supporting Information

**Article title:** Non-invasive assessment of cultivar and sex of *Cannabis sativa* L. by means of hyperspectral measurement

**Authors:** Andrea Matros, Patrick Menz, Alison R. Gill, Armando Santoscoy, Tim Dawson, Udo Seiffert, Rachel A. Burton

**Article acceptance date:** Click here to enter a date.

The following Supporting Information is available for this article:

**Fig. S1** Schematic representation of the field plot map.

**Fig. S2** Mean reflectance spectra from measurements of dioecious *C. sativa* L. plants.

**Fig. S3** Images from the field setting showing developmental differences between the *C. sativa* L cultivars.

**Table S1** Confusion matrix for the differentiation between soil types per cultivar.

**Table S2** Determination of sex from field grown dioecious *C. sativa* L. plants.

**Table S3** Accuracies for the prediction of sex from reflectance spectra.

**Table S4** Calculated F1, precision, and recall values for the models presented in our study.

**Fig. S1** Schematic representation of the field plot map. Plants for eight different cultivars of *Cannabis sativa* L. were grown on a field site of the University of Adelaide, Waite Campus (34°57’58"S 138°38’1"E ), which was either fertilised with regular compost (F) or left un-fertilised as control (C). Acquisition of hyperspectral data was conducted from three leaves (technical replicates) of 15 randomly selected plants per cultivar per condition.

**
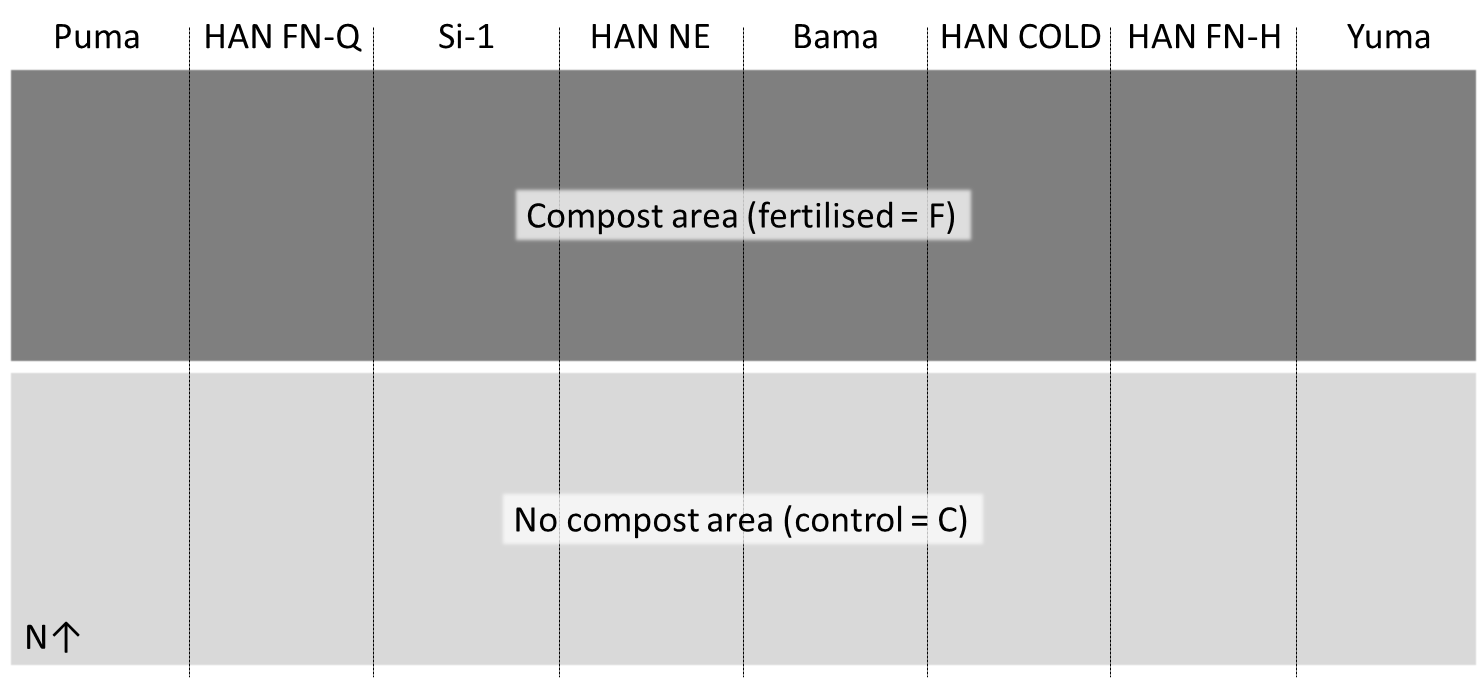
**

**Fig. S2** Mean reflectance spectra from measurements of dioecious *C. sativa* L. plants. Spectra were acquired from three leaves per plant from 15 plants per cultivar from two soil conditions (fertilised – red line and control – blue line). Shown are the mean reflectance spectra of the two soil conditions for each cultivar. Dotted lines indicate the variance range. (a) Yuma, (b) HAN FN-H, (c) HAN COLD, (d) Bama, (e) HAN NE, (f) Si-1, (g) HAN FN-Q, and (h) Puma.

**
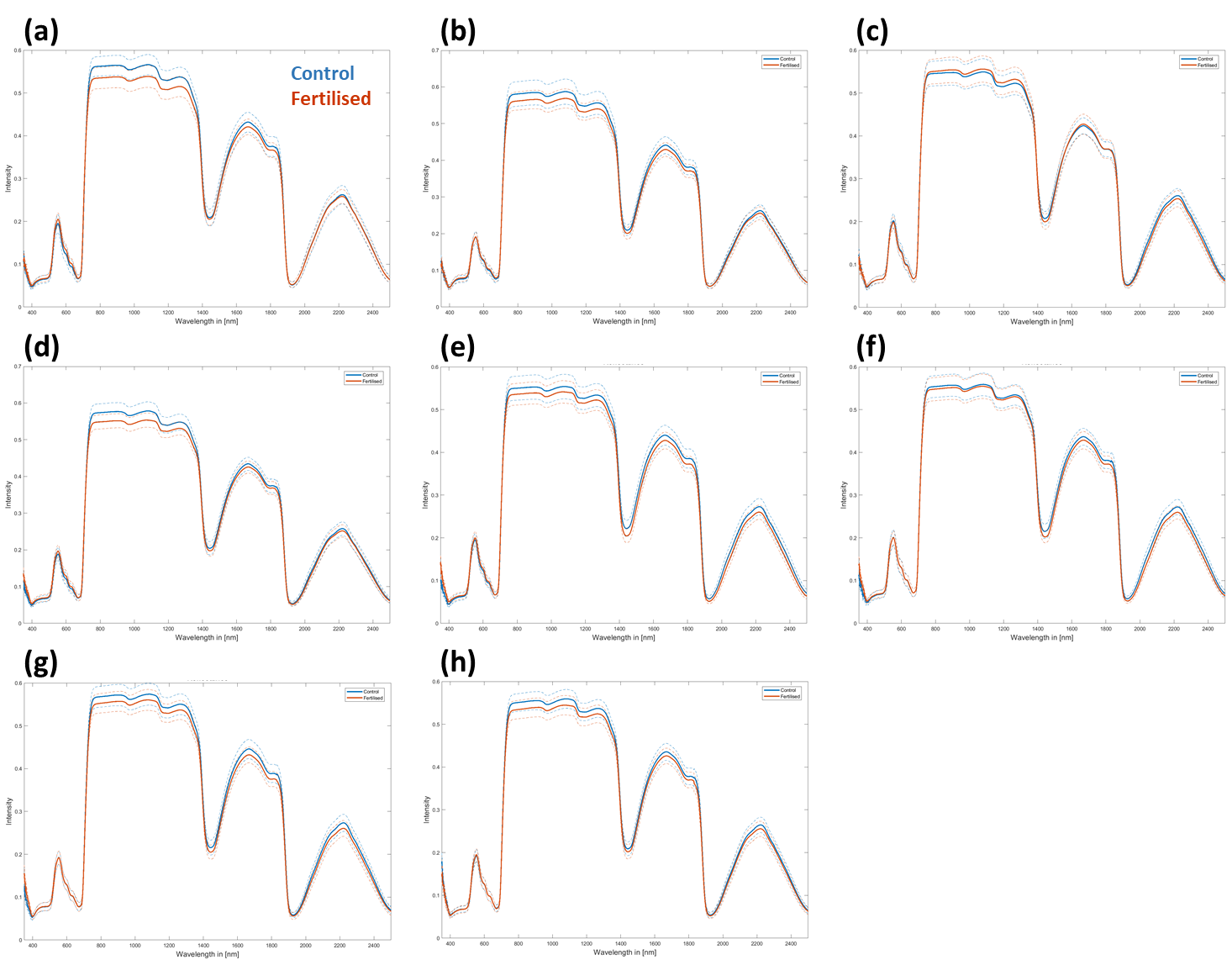
**

**Fig. S3** Images from the field setting showing developmental differences between the *C. sativa* L cultivars. (a) shows the field setting on 05/03/2020 at nine weeks after sowing. *C. sativa* L. cultivars from left to right are Yuma, HAN FN-H, HAN COLD, Bama, HAN NE, Si-1, HAN FN-Q and Puma. (b) and (c) show the field setting on 17/03/2020 at eleven weeks after sowing. *C. sativa* L. cultivars from left to right are in (b) Yuma, HAN FN-H, HAN COLD and Bama, and in (c) HAN NE, Si-1, HAN FN-Q and Puma.

**
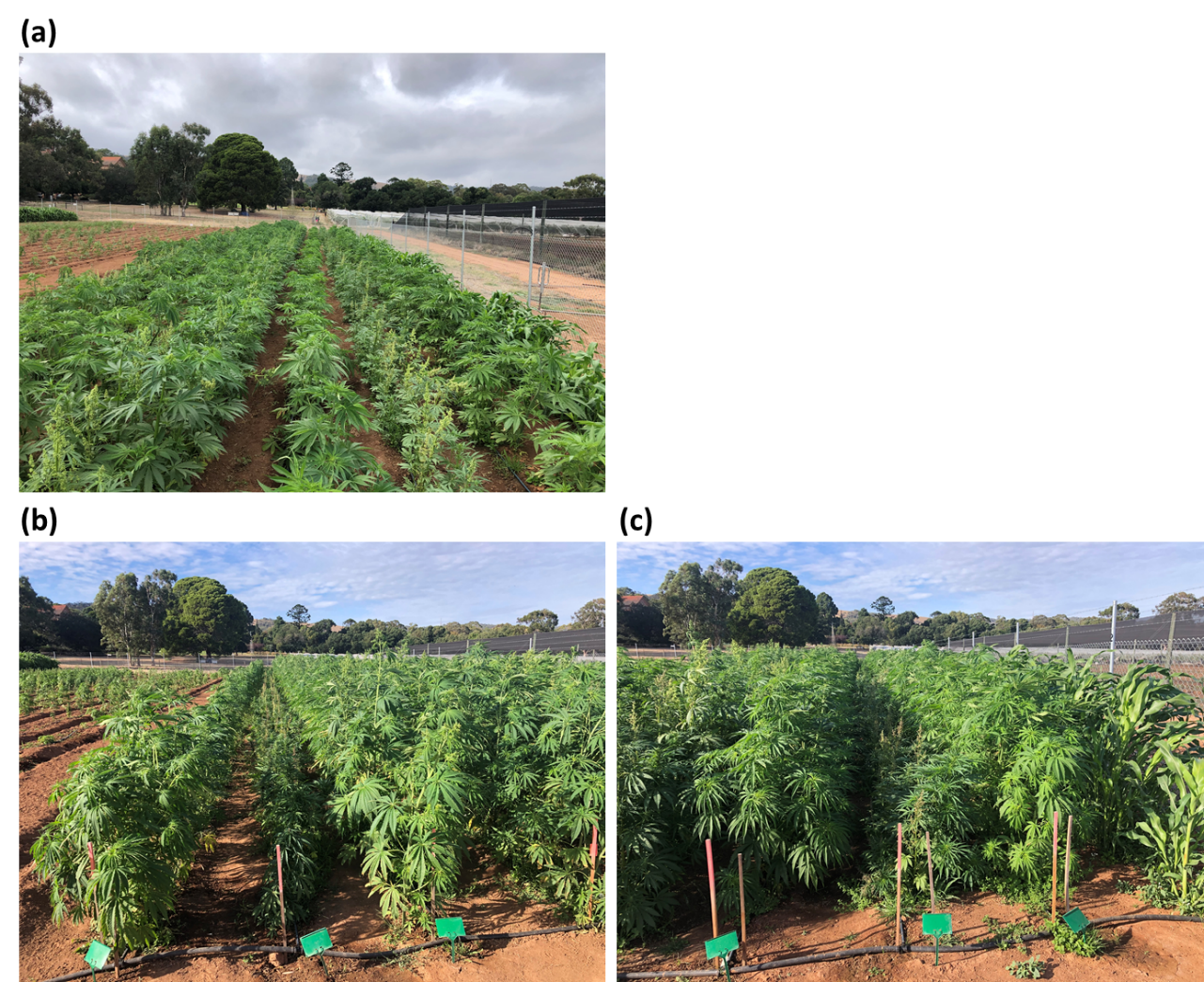
**

**Table S1** Confusion matrices for the differentiation between soil types per cultivar. Correct classification rate by individual measurement applying leave-one-out validation is shown. Shown in brackets are the values for correct classification rate by entire plant applying leave-one-out validation and majority voting. The mean and standard deviation were 1 and 0 for all mathematical models calculated. All spectra of the two soil types were correctly assigned when calculated for the cultivars individually. The best-performing model was an RBF using Euclidean metric and 40 prototypes. Only results for this model are presented.

|  |  |  | **True Class** | |
| --- | --- | --- | --- | --- |
|  |  |  | **Control** | **Fertilised** |
| **Yuma** | **Predicted Class** | **Control** | 45  (15) | 0  (0) |
|  |  | **Fertilised** | 0  (0) | 45  (15) |
| **HAN FN-H** | **Predicted Class** | **Control** | 45  (15) | 0  (0) |
|  |  | **Fertilised** | 0  (0) | 45  (15) |
| **HAN COLD** | **Predicted Class** | **Control** | 45  (15) | 0  (0) |
|  |  | **Fertilised** | 0  (0) | 45  (15) |
| **Bama** | **Predicted Class** | **Control** | 45  (15) | 0  (0) |
|  |  | **Fertilised** | 0  (0) | 45  (15) |
| **HAN NE** | **Predicted Class** | **Control** | 45  (15) | 0  (0) |
|  |  | **Fertilised** | 0  (0) | 45  (15) |
| **Si-1** | **Predicted Class** | **Control** | 45  (15) | 0  (0) |
|  |  | **Fertilised** | 0  (0) | 45  (15) |
| **HAN FN-Q** | **Predicted Class** | **Control** | 45  (15) | 0  (0) |
|  |  | **Fertilised** | 0  (0) | 45  (15) |
| **Puma** | **Predicted Class** | **Control** | 45  (15) | 0  (0) |
|  |  | **Fertilised** | 0  (0) | 45  (15) |

**Table S2** Determination of sex from field grown dioecious *C. sativa* L. plants. Assessment of sex was performed for the measured and labelled (number one to number fifteen) plants in the time between 17/02/2020 and 10/04/2020. Generally higher numbers of female plants were assigned, except for cultivar Si-1 under control conditions and cultivar HAN FN-Q under fertilised conditions. M – male, F – female, and d –dead plant.

**Table S3** Accuracies for the prediction of sex from reflectance spectra. Spectra were measured from leaves before flowering (17/02/2020) for the fifteen individual plants per condition and cultivar. Compared are the classification rates from using either spectra of the individual cultivars or spectra across all cultivars for three datasets: 1) containing all spectra acquired from each cultivar (fertilised and control conditions, 15 plants each with three leaves per plant measured; 90 spectra per cultivar in total), 2) containing all spectra acquired from each cultivar grown under control conditions (15 plants each with three leaves per plant measured; 45 spectra per cultivar in total), and 3) containing all spectra acquired from each cultivar grown under fertilised conditions (15 plants each with three leaves per plant measured; 45 spectra per cultivar in total). Also, the mean classification rate and standard deviation are provided. Significant improvement of classification rates for plants grown under fertilised conditions is indicated by *; paired t-test, p = 0.0046 (spectra from individual cultivar) when compared with dataset 1) and p = 0.0038 (spectra from individual cultivar) when compared with dataset 2). d – indicates dead plants. Prediction accuracies are color-coded: correct assignment of all three spectra (value 1, orange), correct assignment of two out of three spectra (0.667, light orange), false assignment of all three spectra (0, blue), and false assignment of two out of three spectra (0.333, light blue). The underlying best-performing models for the individual classifications were a) an RBF using Euclidean metric and 5 prototypes, b) PLS1 with 20 components, c) MLP with 1 hidden layer and 10 neurons, d) an RBF using Euclidean metric and 15 prototypes (Table 7 in the main manuscript). Respective F1, precision, and recall values are shown in Supplementary Table S4.

**Table S4** Calculated F1, precision, and recall values for all two-class models presented in our study. For discrimination between cultivars (task a), Table 5) no such values are presented as this was an eight-class problem. The underlying best-performing models were as follows:

Differentiation between male and female plants of cultivar Ferimon 12 (Table 4 in the main manuscript): The best-performing model was an RBF using Euclidean metric and 10 prototypes. Differentiation between soil types (Table 6): The best-performing model was an RBF using Euclidean metric and 40 prototypes. Differentiation between sex per cultivar (Table 7): The best-performing models for the individual classifications were a) an RBF using Euclidean metric and 5 prototypes, b) PLS1 with 20 components, c) MLP with 1 hidden layer and 10 neurons, d) an RBF using Euclidean metric and 15 prototypes.

|  |  | **F1-Score** | **Precision** | **Recall/Sensitivity** |
| --- | --- | --- | --- | --- |
| **Differentiation between sex of cultivar Ferimon 12 (Table 4)** |  | 1.000 | 1.000 | 1.000 |
| **Differentiation between soil types (Table 6)** |  | 0.987 | 0.986 | 0.989 |
| **Differentiation between sex per cultivar (Table 7)** | ***1) Fertilised + Control*** |  |  |  |
|  | **Yuma** | 0.696 | 0.784 | 0.625 |
|  | **HAN FN-H** | 0.660 | 0.627 | 0.696 |
|  | **HAN COLD** | 0.786 | 0.950 | 0.671 |
|  | **Bama** | 0.800 | 0.982 | 0.675 |
|  | **HAN NE** | 0.718 | 0.824 | 0.636 |
|  | **Si-1** | 0.698 | 0.815 | 0.611 |
|  | **HAN FN-Q** | 0.557 | 0.611 | 0.512 |
|  | **Puma** | 0.868 | 1.000 | 0.767 |
|  | **Across all** | 0.763 | 0.981 | 0.625 |
|  | ***2) Control Samples only*** |  |  |  |
|  | **Yuma** | 0.571 | 0.476 | 0.714 |
|  | **HAN FN-H** | 0.741 | 0.741 | 0.741 |
|  | **HAN COLD** | 0.697 | 0.852 | 0.590 |
|  | **Bama** | 0.738 | 0.889 | 0.632 |
|  | **HAN NE** | 0.750 | 0.889 | 0.649 |
|  | **Si-1** | 0.558 | 0.571 | 0.545 |
|  | **HAN FN-Q** | 0.680 | 0.630 | 0.739 |
|  | **Puma** | 0.846 | 1.000 | 0.733 |
|  | **Across all** | 0.771 | 1.000 | 0.627 |
|  | ***3) Fertilised Samples only*** |  |  |  |
|  | **Yuma** | 0.870 | 1.000 | 0.769 |
|  | **HAN FN-H** | 0.692 | 0.750 | 0.643 |
|  | **HAN COLD** | 0.829 | 0.879 | 0.784 |
|  | **Bama** | 0.833 | 0.833 | 0.833 |
|  | **HAN NE** | 0.452 | 0.292 | 1.000 |
|  | **Si-1** | 0.880 | 1.000 | 0.786 |
|  | **HAN FN-Q** | 0.000 | 0.000 | 0.000 |
|  | **Puma** | 0.880 | 1.000 | 0.786 |
|  | **Across all** | 0.827 | 0.940 | 0.738 |
